# Nanostructuring of Palladium with Low-Temperature Helium Plasma

**DOI:** 10.3390/nano5042007

**Published:** 2015-11-25

**Authors:** P. Fiflis, M.P. Christenson, N. Connolly, D.N. Ruzic

**Affiliations:** Department of Nuclear, Plasma and Radiological Engineering, Center for Plasma Material Interactions, University Illinois at Urbana-Champaign, Urbana 61801, IL, USA; E-Mails: mpchris2@illinois.edu (M.P.C.); connlly2@illinois.edu (N.C.); druzic@illinois.edu (D.N.R.)

**Keywords:** palladium, nanotendrils, helium bubbles, catalysis

## Abstract

Impingement of high fluxes of helium ions upon metals at elevated temperatures has given rise to the growth of nanostructured layers on the surface of several metals, such as tungsten and molybdenum. These nanostructured layers grow from the bulk material and have greatly increased surface area over that of a not nanostructured surface. They are also superior to deposited nanostructures due to a lack of worries over adhesion and differences in material properties. Several palladium samples of varying thickness were biased and exposed to a helium helicon plasma. The nanostructures were characterized as a function of the thickness of the palladium layer and of temperature. Bubbles of ~100 nm in diameter appear to be integral to the nanostructuring process. Nanostructured palladium is also shown to have better catalytic activity than not nanostructured palladium.

## 1. Introduction

Experiments at several different institutions have observed the growth of tungsten nanostructures under exposure to helium plasmas while investigating the viability of tungsten for high heat flux components in nuclear fusion reactors [1,2]. While these structures are potentially fatal to the fusion plasma when grown [3], they do exhibit characteristics that could be exploited in other applications. A high porosity, a low density of about 10% of the bulk material, large surface area, increased emissivity, and decreased reflectance are all properties of the nanostructured surface [4,5,6,7]. The nanostructures are produced by prolonged exposure to a flux of helium ions while the tungsten is at an elevated temperature. Several studies have investigated the similar formation of nanostructures on metals other than tungsten. Exposure to fluxes of helium in excess of 10^20^ m^−2^·s^−1^ at a temperature approximately between 30% and 50% of the melting temperature yields several different nanostructures, *i.e.*, cones/pillars have been observed on copper [8], fuzz on molybdenum and tungsten [5], and roughening of the surface on titanium [8]. Palladium is widely used as a catalyst in a variety of different chemical reactions; and because catalytic activity scales linearly with the surface area of the catalyst, experiments were undertaken to investigate the nanostructuring of palladium under irradiation by helium ions. To this end, several palladium substrates were exposed at elevated temperatures to helium ions from a helicon plasma source. The dependence of the formed nanostructures on temperature and sample geometry are described herein.

## 2. Experimental Section 

**Figure 1:**
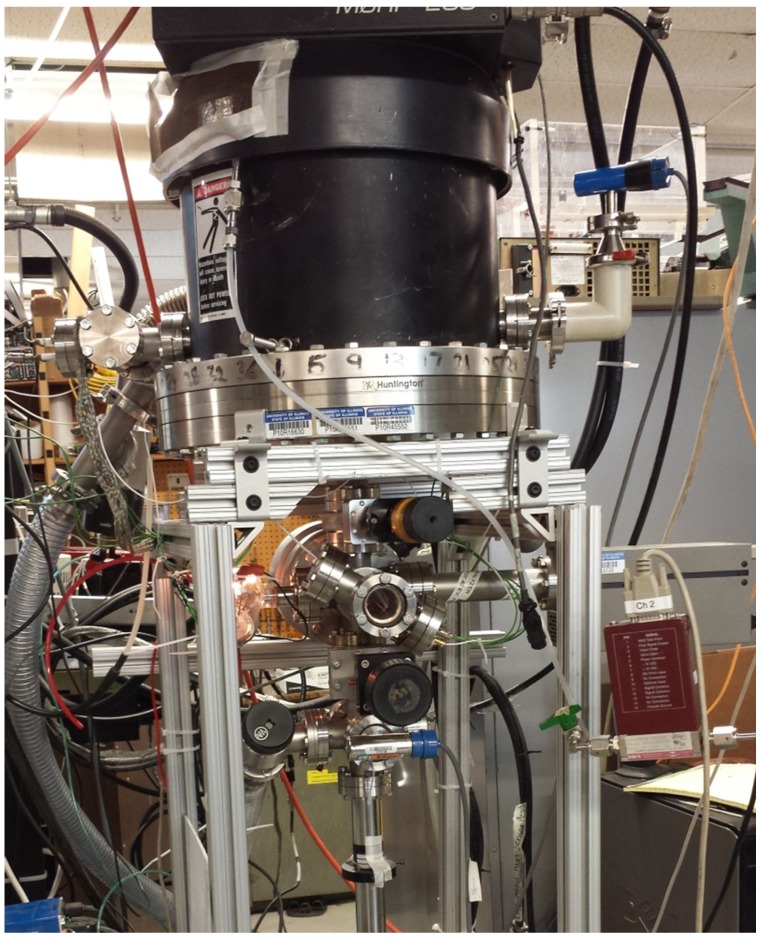
Photo of exposure chamber showing MORI (Trikon Technologies, Newport, UK) automated matching network, exposure volume, load lock gate valve, and transfer arm for introducing samples without breaking vacuum.

Palladium samples (Alfa Aesar 99.9%, Ward Hill, MA, USA) were exposed inside of a commercial grade helium helicon source (MORI 200, Trikon Technologies, Newport, UK) [9]). The plasma conditions for the experiments described herein were generated with an RF power of 700 W, a magnetic field of 120 G, and a background helium pressure of 100 mTorr as read by a convectron gauge (Granville Phillips 375, MKS Instruments, Andover, MA, USA). A photo of the experimental chamber can be seen in Figure 1. The resulting plasma density is 1 × 10^18^ m^−3^ with an electron temperature of 4 eV diagnosed with an RF-compensated Langmuir probe [10] in the region where the sample was placed. The palladium was supported via a copper sample holder, which suspended the sample in the plasma. The sample was biased to negative 40 V with respect to plasma potential such that the incoming helium ion flux had an energy of 40 eV and a flux of 2.5 × 10^21^ m^−2^·s^−1^. Sample temperature was achieved merely by heating of the sample via the incoming ion flux. Regulation of the temperature, however, was achieved by adjusting the area of the sample in direct contact with the copper sample holder, thereby controlling conduction losses. It should be noted that the centerline density of the plasma is constant to within measurement error over the range of sample placements, so adjustment of the sample relative to the copper sample holder did not change the flux to the target. Temperatures were not directly measured, but rather computed via an experimentally calibrated finite difference model which balances input energy from helium ion irradiation and losses via conduction and radiation [11]. Scanning electron microscopy (Hitachi S4700, Tokyo, Japan) was performed on the exposed samples. Four different geometries were tested; a plate of palladium 1 cm × 2 cm × 0.5 mm, a wire 0.5 mm diameter × 20 cm in length, and two thin films deposited on 25 mm × 25 mm glass substrates with thicknesses of 300 nm (evaporation coating) and 30 nm (magnetron sputter coating).

## 3. Results and Discussion

### 3.1. Palladium Nanostructuring as a Function of Temperature

**Figure 2:**
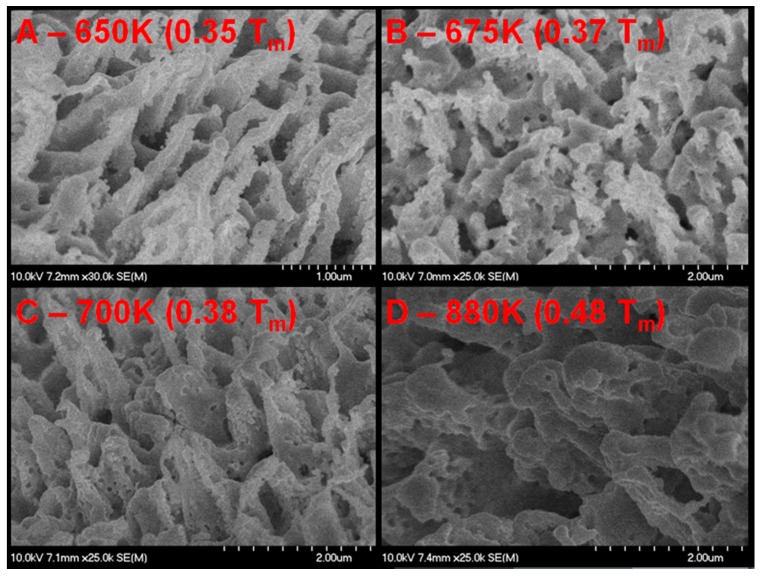
Scanning electron microscope (SEM) micrographs of palladium surface (0.5 mm diameter wire sample) after exposure to helium plasma at elevated temperature. The flux to each area is identical, the only changed variable is temperature (noted in the upper left corner of each micrograph both absolute and as a fraction of the melting point of palladium).

**Figure 3:**
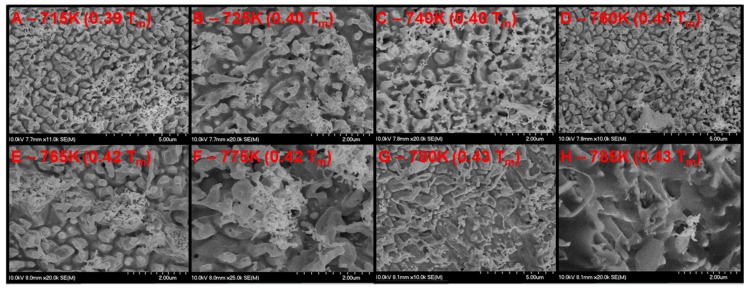
SEM micrographs of palladium surface (0.5 mm plate sample) after exposure to helium plasma at elevated temperature. The flux to each area is identical, the only changed variable is temperature (noted in the upper left corner of each micrograph both absolute and as a fraction of the melting point of palladium). Secondary electron collection performed at a tilt angle of 0° with respect to the surface normal.

**Figure 4:**
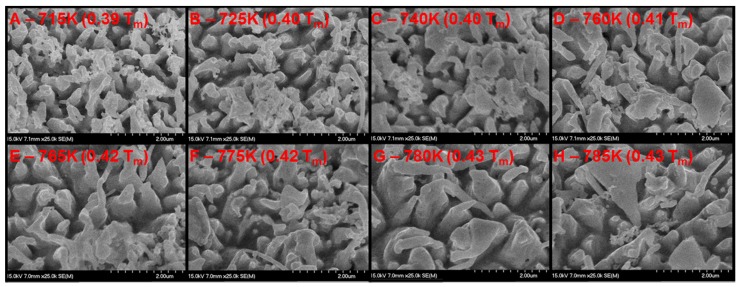
SEM micrographs of palladium surface (0.5 mm plate sample) after exposure to helium plasma at elevated temperature. The flux to each area is identical, the only changed variable is temperature (noted in the upper left corner of each micrograph both absolute and as a fraction of the melting point of palladium). Secondary electron collection performed at a tilt angle of 40° with respect to the surface normal.

**Figure 5:**
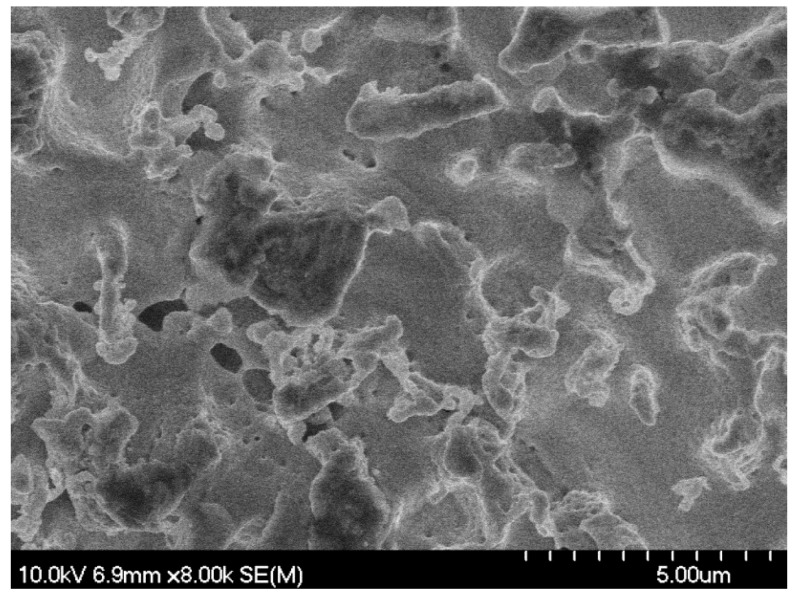
SEM micrograph of palladium surface (0.5 mm diameter wire sample) after exposure to helium plasma at 900 K, only a couple tendrils are visible as the annealing rate of the tendrils begins to exceed the rate of growth.

Scanning electron microscope (SEM) micrographs of the exposed wire and plate are shown in Figure 2, Figure 3 and Figure 4, respectively. Figure 3 is a series of top down (0° from normal) micrographs of the plate; Figure 4 is a series of micrographs of the plate and has a tilt of 40° from normal introduced to the sample. From these micrographs it can be seen that exposure of palladium at elevated temperatures to fluxes of helium ions forms a series of pillars or tendrils from the surface. Energy dispersive X-ray Spectroscopy (EDX) analysis of these tendrils confirms that they are palladium. These tendrils are 350 ± 100 nm in diameter, 1000 ± 250 nm in height, and possess an areal density of approximately 1.5 tendrils/µm^2^. It can also be seen from the difference between the 0° and 40° micrographs of the plate that these tendrils grow normally to the surface. The tendrils are also mostly straight at temperatures less than 700 K, and in excess of 700 K the structures begin to bend and fold. As the temperature increases beyond this (>775 K), the nanostructures begin to increase in diameter (500 ± 100 nm), but not length. The areal density of tendrils decreases to approximately 1 tendril/µm^2^. As the temperature exceeds 850 K, the nanostructures appear to begin to anneal back into the bulk, resulting in very rounded and thick tendrils at 880 K (see Figure 2), and full disappearance of tendrils by 900 K (Figure 5). The nanostructuring process appears to be bubble driven, similar to the growth of tungsten nanostructured “fuzz” which is predicted to grow under certain conditions in the divertor region of fusion reactors [12]. However, while the bubbles that drive nanostructuring in tungsten are approximately 10 nm in diameter, the pits observed in the surface of the nanostructured palladium (attributed to bubble bursting at the surface similar to tungsten [11]) are 75 ± 25 nm in diameter at temperatures less than 750 K. At temperatures above 750 K, these pits swell in size to 110 ± 30 nm in diameter. Normalizing two characteristic parameters of the nanostructures to the pit diameter draws striking parallels between tungsten and palladium nanostructuring. The ratio of tendril diameter to pit diameter in tungsten is approximately 3 to 4 [11]. Similarly, the ratio of tendril diameter to pit diameter in palladium is also 3 to 4. Additionally, the separation distance between individual tendrils is approximately 7 to 11 times the diameter of the pits in tungsten [11]. With a tendril separation distance of 800 ± 150 nm, palladium nanostructures have a ratio of tendril separation distance to pit diameter in the exact same range. This is very indicative of a similar bubble mechanism driving the growth of fuzz tendrils, whereby bubbles created in the bulk of the material effectively rise to the surface or grow and thin the material above them, subsequently rupturing. As more bubbles impact the surface, hills and valleys start to form stochastically. Bubbles then are more likely to connect to a valley rather than a hill by virtue of shorter path length, and as a result nanostructures grow. Characteristics such as the ratio of tendril diameter or tendril separation distance to the pit diameter fall out of a simple Monte Carlo model implementing only the assumption that bubbles are more likely to rupture at a valley than a hill [11]. Since the model is independent of material properties, any nanostructuring via the same mechanism will show similar tendril to pit diameter ratios and tendril separation to pit diameter ratios, as is seen here. Much like tungsten nanostructuring via helium plasma bombardment, palladium nanostructuring appears to have a window of temperature for which it can grow tendrils. Tungsten fuzz grows within the temperature range of 1000–2000 K (0.27–0.54 *T*_m_) [13]. Palladium nanostructuring in the experiments described herein occurred at 650 K (0.33 *T*_m_) and 880 K (0.48 *T*_m_) as well as several other intermediate temperatures and was bracketed by a lack of nanostructuring formation at 500 K (0.27 *T*_m_) and 900 K (0.49 *T*_m_). When normalized to the melting point of the material, the active temperature range for the bubble driven nanostructuring appears to be very similar.

### 3.2. Palladium Nanostructuring versus Palladium Thickness

**Figure 6:**
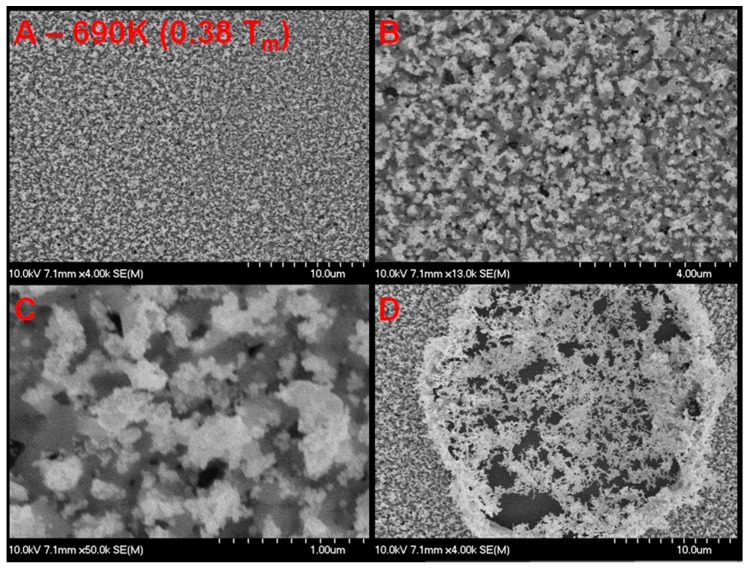
SEM micrograph of palladium surface (300 nm thin film deposited on SiO_2_) after exposure to helium plasma at elevated temperature. (**A**), (**B**), and (**C**) are different resolutions of the same location showing growth of tendrils and voids that appear to penetrate down to the SiO_2_ substrate. Tendrils approximately the same diameter as those observed on bulk Pd samples are observed. Pits of similar diameter are also observed. (**D**) shows an area of the palladium film where the helium plasma has eroded through the palladium film to the substrate with very thin tendrils of Pd stretching across.

**Figure 7:**
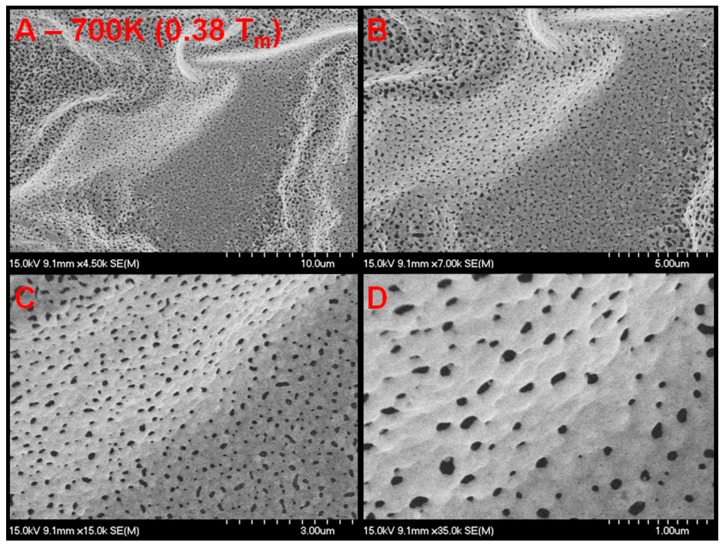
SEM micrograph of palladium surface (30 nm thin film deposited on SiO_2_) after exposure to helium plasma at elevated temperature. Figure 5A–D are different resolutions of the same location showing growth no tendril growth, but a significant amount of voids. These voids are of a diameter greater than the pits observed in the bulk and 300 nm film samples. Large wrinkles appear evident in the film. It appears as though formation and growth of bubbles within the 30 nm thick film rupture the film without being able to build upon each other and grow nanostructures.

Many applications of palladium nanostructuring revolve around the increased surface area to volume ratio (*i.e.*, improving the catalytic activity for a given weight of palladium). As a result of this, the ability to grow nanostructures on thin films of palladium is very desirable as this would further increase the surface area to volume ratio of an amount of palladium with an already large surface area to volume ratio. There, however, was thought to be a minimum thickness at which the palladium would no longer nanostructure because the bubbles necessary to drive the growth of palladium nanostructures were approximately 100 nm in diameter and could therefore not grow to full size in very thin films. As thick substrates of 0.5 mm thickness and diameter had already been tested, two thin films (of thickness 300 and 30 nm) were investigated to see the structures that would form. SEM micrographs of each sample can be seen in Figure 6 and Figure 7, respectively. Nanostructure growth for the 300 nm film appears to be again bubble driven. Tendrils of diameter 320 ± 100 nm are apparent and so are pits of 75 ± 15 nm diameter. These are commensurate with the diameters of the tendrils and pits of the bulk samples at the same temperature of exposure. The tendrils are also very straight, much like the tendrils observed in the bulk samples at temperatures <700 K. This implies the bubble formation depths and subsequent loop punching to the surface occurs at depths of less than 3 bubble diameters, much like the formation of helium bubbles within tungsten which drive nanostructuring [11]. As the thickness of the film is reduced below that of the bubble diameter, formation of full helium bubbles to drive nanostructuring is suppressed. Instead, it appears as though formation and growth of bubbles within the 30 nm thick film rupture the film without being able to build upon each other and grow nanostructures. This results in a series of pits in the surface, but no vertical growth of nanostructures. These pits are of diameter 130 ± 35 nm, which is larger than those observed in the 300 nm and bulk samples. Wrinkles are also evident in the palladium film which is indicative of delamination of the palladium film from the SiO_2_ substrate. The palladium film was deposited via magnetron sputtering at room temperature and due to residual tensile stresses in the film, once it became delaminated, it wrinkled.

### 3.3. Catalysis with Nanostructured Palladium

It has been suggested that the increased surface area of nanostructured palladium could provide greatly enhanced catalytic activity. To investigate this hypothesis, a comparison was made between identical plates, one not nanostructured and the other nanostructured under identical conditions to the plate described above. This comparison of catalytic properties was carried out using a reduction reaction, which modified cyclohexene into cyclohexane through the syn addition of two hydrogens using the palladium pieces as the catalysts for the reaction [14]:

C_6_H_10_ + H_2_ → C_6_H_12_

The catalytic properties of each of the palladium samples, both smooth and nanostructured, were compared to the industrial palladium catalytic standard of palladium absorbed onto a carbon surface [15]. This standard is widely accepted as the best way to increase surface area for catalytic reactivity.

Alkenes are reduced to alkanes through a multi-body process, where the palladium or other catalyst acts as an activation site for the reaction. Following the Horiuti-Polanyi mechanism [14], hydrogen first dissociatively chemisorbs to the bonding site on the palladium surface, but only if the hydrogen molecule has its axis parallel to the surface of the palladium crystal [16]. For this reason, hydrogen is often found in excess in these reactions. Cyclohexene, present in the reaction in liquid form, dissociatively bonds to the surface of the palladium in much the same way as the hydrogen molecule, where the pi bond in the double bond between the carbons is broken. The carbons then bond to the palladium surface. The dissociated hydrogen atoms then bond to the free sites on the carbon atoms currently bonded to the palladium surface and the molecule leaves the palladium surface as an alkane since alkanes are not strongly adsorbed on the surface. For a single reaction on a simple palladium surface, this leads to a significant reduction in the activation energy, making the reaction feasible for scale-up.

**Figure 8:**
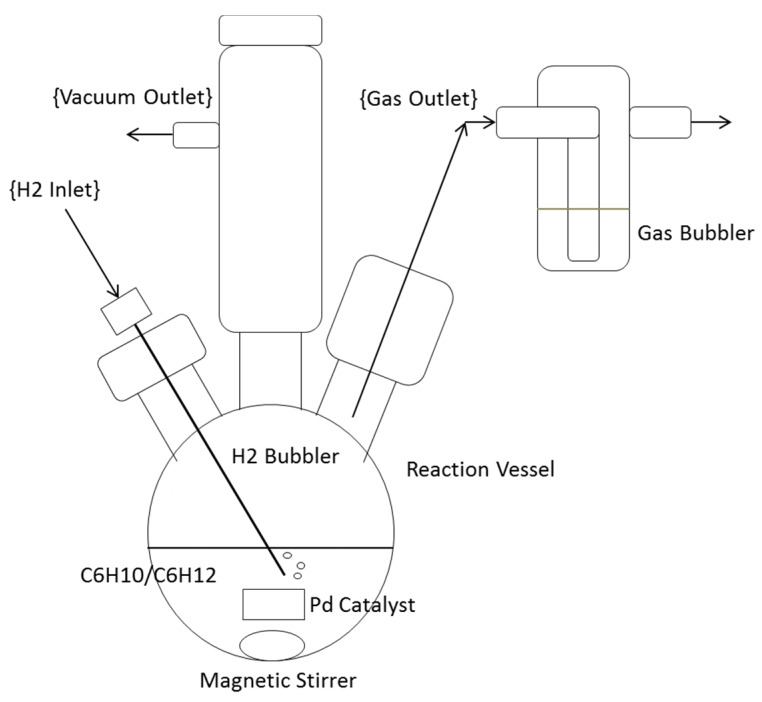
A block diagram of the palladium-catalyzed hydrogenation reaction vessel, including the hydrogen gas inlet, the vacuum cylinder outlet, the magnetic stirrer, and the gas bubbler used to qualitatively determine the flow rate of the hydrogen through the reactant volume.

All reactions were carried out with the primary reactant, cyclohexene, in liquid phase. A block diagram of the experimental setup can be seen in Figure 8. The reaction vessel was a 500 mL, three-neck round bottom flask connected to a vacuum line on one neck, a gas bubbler on the second neck, and stoppered on the third neck. Prior to closing off the flask, the catalytic samples were weighed and added, along with a magnetic stir rod. The reaction chamber was then sealed. The chamber was then purged with argon and evacuated three separate times to ensure atmospheric purity. 150 mL of cyclohexene was added through the stopper using a syringe. A second syringe connected to a hydrogen line was then inserted into the cyclohexene liquid in order to bubble into the flask an excess of hydrogen gas, as noted by the bubbler. Before hydrogen was added, a control sample was taken. Samples were then taken using a microsyringe at 30 min intervals for 180 min, while the hydrogen was bubbled through the reactants and the stir rod agitated the reactants. This was done to test the reaction rate for each of the catalyst types. All samples taken were 100 µL in volume and were added to NMR sample tubes along with 600 µL of deuterated chloroform (CDCl_3_), which were mixed through inversion.

**Figure 9:**
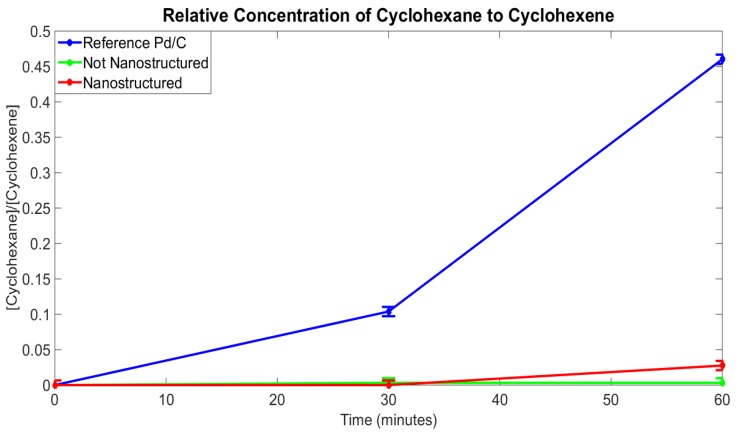
A plot of the yield measured as the ratio of the concentration of the cylcohexane to cyclohexene. This ratio is based on the ratios of areas under the curve of the cyclohexane 1.4 ppm peak and the mean of the intensities of the three cyclohexene peaks seen at 1.2 ppm, 1.7 ppm, and 2.0 ppm in the NMR scans, which are characteristic for the respective compounds and are normalized to the deuterated chloroform standard. These ratios were also taken as a function of time to, not only observe the effectiveness of each catalyst type, but also how the kinetics of the reaction compare with each catalyst type.

The catalytic capability of the different palladium types was measured by determining the ratios of the areas under the typical cyclohexene peaks to the typical cyclohexane peaks seen in Nuclear magnetic resonance (NMR) spectra. A Varian Unity Inova NMR spectroscopy machine [17,18] was used to make the measurements, and each scan used a spectral frequency of 399.74 MHz, with 1 transient at an acquisition time of 4.096 seconds. The scans were performed using one-dimensional proton NMR at a 45 degree pulse width. Ratios were taken between areas under the 1.4 ppm cyclohexane peak and the mean of the areas under the 1.2 ppm, 1.7 ppm, and 2.0 ppm cyclohexene peaks, normalized to the deuterated chloroform standard. Figure 9 shows the results of the catalytic conversion over time for the different catalyst types, indicating that the fuzzed sample is greatly improved in conversion over the non-fuzzed sample.

## 4. Discussion and Conclusions

A variety of palladium samples were exposed to a flux of 40 eV helium ions at temperatures between 0.3 and 0.5 *T*_m_. Samples of bulk palladium (*i.e.*, wire and plate) showed evidence of bubbles of approximate diameter 100 nm and tendrils of approximate diameter 350 nm. The nanostructuring growth mechanism appears to be similar to that of tungsten, with an active temperature range similar to that of tungsten after normalization to the melting point. However, the diameter of the bubbles is much larger than that of those observed in tungsten. Previous studies of exposure of different metals to energetic helium fluxes at elevated temperatures have suggested that the nanostructuring process is heavily dependent on crystal structure [8]. Body centered cubic (bcc) crystals, such as tungsten, molybdenum, and tantalum, show very similar nanostructures in both size and morphology. Palladium is a face centered cubic (fcc) material, and therefore, will nanostructure differently than the bcc tungsten. However, since the ratio of tendril diameter to pit diameter as well as the ratio of inter-tendril separation to pit diameter is the same for both tungsten and palladium, it is highly probable that the mechanism for the formation of the nanostructures is the same. The difference in bubble size then is the biggest driver in the difference in observed morphology.

Nanostructuring the palladium plate resulted in a large increase in catalytic activity beyond that of the non-nanostructured sample. It should, however, be noted that the reference Pd/C catalyst outperformed both of the fuzzed and non-fuzzed samples due to the very large surface area provided by the activated carbon. Mechanical removal of these nanostructures from the surface of the palladium to produce a finely nanostructured powder catalyst may increase the catalytic activity beyond that of the standard. Alternatively, nanostructuring of other geometries with a helium plasma may offer advantages in systems where filtration of powder catalysts would be impractical and conventional methods of surface roughening, such as sand blasting, would be too violent on fragile catalyst geometries. Nanostructuring by helium plasma could also be performed atop a sand blasted layer to further increase the surface area.
